# Physical Activity in Patients With Chronic Kidney Disease: The Effects of Caregivers and Residential Factors

**DOI:** 10.7759/cureus.74525

**Published:** 2024-11-26

**Authors:** Marieta Theodorakopoulou, Fotini Iatridi, Io Giannopoulou, Eleni Karkamani, Artemios G Karagiannidis, Erasmia Sampani, Konstantina Dipla, Pantelis Sarafidis, Afroditi Boutou

**Affiliations:** 1 Department of Nephrology, Aristotle University of Thessaloniki, Thessaloniki, GRC; 2 Department of Medicine, Department of Physical Education and Sports Science, University of Thessaly, Larissa, GRC; 3 Department of Physical Education and Sports Science, Aristotle University of Thessaloniki, Serres, GRC; 4 Department of Respiratory Medicine, Hippokration Hospital, Thessaloniki, GRC; 5 Department of Medicine, Department of Physical Education and Sports Science, University of Thessaly, Thessaloniki, GRC

**Keywords:** carers, chronic kidney disease (ckd), exercise, physical activity, residential factors

## Abstract

Introduction

Physical inactivity is common in chronic kidney disease (CKD) patients; several patient- and disease-related factors are linked to a sedentary lifestyle, but social and environmental influences remain unexplored. This study evaluates the level of physical activity in patients with CKD and investigates the associations with caregivers' physical activity levels, characteristics of the residential environment, and objective measures of exercise capacity.

Methods

Eighty CKD patients (20 per CKD stage 2-4) were included; patients and their carers filled out the International Physical Activity Questionnaire (IPAQ), questionnaires about residential environment and past exercise habits. The CKD patients also performed a 30-sec sit-to-stand-test (30-STS) as a measure of exercise capacity.

Results

According to the IPAQ-score, CKD patients were largely inactive (only 10% reported health-enhancing activity levels); no differences were found between CKD stages. The 30-STS was also impaired, and the number of repetitions significantly decreased with advancing CKD stages (14.2±2.9 vs. 12.6±5.4 vs. 10.4±3.3 vs. 10.5±3.5; p=0.008). Carers were also highly inactive but still had higher physical activity compared to patients (moderate intensity metabolic equivalents (METS) p=0.03, vigorous intensity METS p=0.053); no correlation between carers' and patients' physical activity was noted (p=0.702). Patients' physical activity was associated with residential factors: living in a detached house compared to an apartment (p=0.013 for moderate intensity and p=0.017 for vigorous METS), having a garden (p=0.019 for moderate intensity and p=0.022 for vigorous METS) and accessing house by stairs (p=0.037 for vigorous METS), were associated with higher physical activity.

Conclusions

Physical activity was significantly reduced in CKD patients, irrespectively disease stage. Carers were also highly inactive, but no association was found between the patients' and carers' daily activities. Residential characteristics were the only factors found to be associated to patients' physical activity levels. Personalized, supportive interventions are required, taking into account the patients' social support and microenvironment, in order to increase the physical activity level in this population.

## Introduction

Chronic kidney disease (CKD) is a major global health concern; current estimates indicate that over 850 million individuals worldwide are affected [1], with a global prevalence ranging from 8 to 16% [2]. According to Kidney Disease Improving Global Outcomes (KDIGO), CKD is classified in five stages according to GFR: stage 1 is kidney damage with normal or high glomerular filtration rate (GFR) ≥ 90mL/min/1.73m2; stage 2 is mildly decreased GFR (60-89 mL/min/1.73m2); stage 3a is mildly to moderately decreased GFR (45-59 mL/min/1.73m2); stage 3b is moderately to severely decreased GFR (30-44 mL/min/1.73m2); stage 4 is severely decreased GFR (15-29 mL/min/1.73m2); and stage 5 is kidney failure (GFR<15 mL/min/1.73m2 or dialysis). Stages 1 and 2 require the presence of evidence of kidney damage (eg. albuminuria, urine sediment abnormalities, persistent hematuria, electrolyte and other abnormalities due to tubular disorders, abnormalities detected by histology, structural abnormalities detected by imaging, and history of kidney transplantation). Due to the increased rates of traditional cardiovascular risk factors such as hypertension, diabetes mellitus, and obesity [3], CKD currently lists among the fastest-growing global causes of death and is expected to become the fifth global cause of death by 2040 [2]. Beyond high morbidity and mortality rates, CKD is associated with substantial changes in physical and mental functioning, leading to a marked reduction in quality of life [4,5].

Physical activity is any physical movement produced by skeletal muscle that requires energy expenditure [6]. Published data indicate that physical inactivity is common amongst the CKD population and end-stage renal disease patients on renal replacement therapy, although there is evidence that exercise is safe and beneficial in this group of patients [7-9]. It has been shown that even small increases in physical activity levels can improve exercise tolerance and cardiovascular responsiveness and improve quality of life in patients with CKD [10] while, on the other hand, inactivity is associated with decreased renal function, increased re-admission and all-cause mortality in CKD [11].

Multiple factors (patient-related, disease-related, and environment-related) may contribute to the high prevalence of physical inactivity and sedentary lifestyles in CKD patients [12]. Patient-related factors include older age and a higher number of comorbidities, while female gender has also been associated with lower physical activity [13]. Disease-related factors include fatigue, polypharmacy, uremia, chronic inflammation, insulin resistance, metabolic acidosis, anemia, chronic kidney disease-mineral and bone disorder (CKD-MBD), sarcopenia, protein-energy wasting, and endothelial dysfunction [13,14]. These changes lead to physical inactivity, deconditioning, depression, feelings of tiredness, social isolation, and finally, a substantial decrease in the quality of patients' lives [15,16]. Thus, the latest Kidney Disease Improving Global Outcomes Clinical Practice Guideline for the Evaluation and Management of Chronic Kidney Disease lifestyle section recommends that individuals with CKD engage in physical activity for at least 30 minutes per day, five times per week [17,18].

While several patient- and disease-related factors have been linked to a sedentary lifestyle, many social and environmental influences remain unexplored. Studies indicate that the attitudes of healthcare providers, including physicians and nurses, significantly impact the physical activity levels of CKD patients, particularly those in the end stages [19]. However, there is currently no research examining the potential influence of family members' or caregivers' attitudes toward physical activity on these patients. Additionally, while living conditions and residential environments have been shown to influence physical activity levels in individuals with other chronic conditions, such as chronic obstructive pulmonary disease (COPD), this relationship remains unexplored in CKD patients [20]. Thus, the aim of this study is to evaluate for the first time the level of physical activity in patients with CKD across the disease spectrum and investigate its potential associations with caregivers' physical activity levels, the characteristics of their residential environment, and with objective measures of exercise capacity, a 30 second sit-to-stand test.

## Materials and methods

Study design and participants

This cross-sectional study enrolled adult (>18 years) patients with CKD stages 2 to 4 (CKD-EPI eGFR: <90 and ≥15mL/min/1.73m2) followed at the outpatient clinic of the First Department of Nephrology, Hippokration Hospital, Thessaloniki, Greece. Inclusion criteria were: 1) adult patients (>18 years) with CKD stages 2 to 4 (CKD-EPI eGFR: <90 and ≥15mL/min/1.73m2), 2) attendance of the patient with a member of his family with whom he resides (partner or caregiver), 3) informed consent from both patient and caregiver to participate in the study. Exclusion criteria were: 1) the presence of end-stage kidney disease (ESKD), 2) severe mental disorders that make informed consent to be obtainment impossible from either one of the participants, 3) inability of either one of the participants to fully understand the Greek language in order to complete the questionnaires, and (4) significant orthopedic or muscle disorders in patients with CKD, which would preclude the sit-to-stand test from being performed. All procedures were performed according to the Declaration of Helsinki (2013 Amendment). The study protocol was approved by the Institutional Review Board of the Hippokration Hospital (number 365/30.07.2020).

The primary outcome of the study was to assess the levels of daily physical activity in patients with CKD stages 2-4 and examine the potential correlation between the levels of physical activity of the patients and those of their partners or caregivers (carers). Secondary outcomes included exploring the potential association between patients' physical activity and exercise capacity levels and identifying residential factors associated with physical activity.

Study procedures and data collection

A consecutive population of CKD patients who visited the outpatient clinic within 6 months was included in the study. In order all disease stages to be equally represented, patients were included in groups of four; every time an eligible patient along with their carer was identified, three other consecutive patients (and their carers) for the rest of the stages were identified and were all included in the study. Participating patients were then scheduled to visit the outpatient's clinic after a 12-hour fasting and without using caffeine, alcohol, or tobacco; all participants were instructed to receive their standard medication. Baseline demographic and anthropometric characteristics, past medical history, and concomitant medication were re-recorded. A physical examination was performed, and venous blood samples were collected for routine laboratory tests and accurate re-estimation of the glomerular filtration rate.

During their visit, all patients had to complete: a) a questionnaire including information about demographics, level of education, and medical and smoking history. b) The International Physical Activity Questionnaire (IPAQ, short form) - a nine-item, self-completed instrument used for the evaluation of physical activity by summing up vigorous, moderate-intensity, and walking (low-intensity) physical activity over the previous seven-day period and generating a total physical activity score, expressed in metabolic equivalents-minutes per week (MET min/wk). Based on the IPAQ scoring procedure, physical activity status is classified into three categories (physical activity classes): 1) low physical activity (inactive subjects; total physical activity score < 600 MET min/wk); 2) moderate physical activity; and 3) high physical activity (health-enhancing physically active subjects; i.e., total IPAQ score ≥ 3000 MET min/wk or vigorous physical activity score ≥ 1500 MET min/wk) [21,22]. A Greek version of IPAQ-short has been previously used in the Eurobarometer survey conducted by the European Union [21]. And c) a questionnaire regarding the patient's residential environment, i.e., type of housing, accessibility, and use of transport, as well as information regarding participation in any systematic exercise or rehabilitation program in the past or present.

Subsequently, patients performed the 30-second sit-to-stand test (30-STS). The 30-STS is a measure of performance in standing up and sitting down as fast as possible in a chair with a backrest [23] and has been previously found to be a reliable tool associated with exercise capacity in CKD patients [24]. The total number of repetitions for 30 seconds was recorded, as well as arterial oxygen saturation (SpO2) via a pulse oximeter and systemic blood pressure (BP) via a cuff sphygmomanometer just before the beginning and at the end of the test. BP measurements were conducted according to guidelines.

The patient's carer had to fill in a separate room: a) the IPAQ physical activity questionnaire, and b) a questionnaire including information regarding exposure to any systematic exercise in the past or present.

Statistical analysis

Continuous variables are reported as mean±standard deviation (SD) or median [interquartile range] according to the normality of distribution tested with the Shapiro-Wilk test. Categorical variables are presented as absolute frequencies and percentages (n, %). Comparison between >2 groups for continuous variables was performed with one-way ANOVA or the Kruskal-Wallis test, according to the normality of distribution. For comparisons between two groups, the independent t-test or the Mann-Whitney test, where applicable, was applied. Chi-squared or Fisher's exact test was used for comparisons of categorical variables. The Pearson correlation coefficient was utilized to explore the correlation between continuous variables. We also performed univariate and multivariate linear regression to evaluate the association of various demographic and clinical characteristics with either IPAQ score or STS repetitions examined as dependent variables. A p-value of <0.05 (two-tailed) was considered statistically significant for all comparisons. Statistical analysis was performed with Statistical Package for Social Sciences version 22 (IBM Inc., Armonk, New York).

## Results

Participant characteristics

Baseline demographic, clinical, and residential characteristics of the study population are presented in Table 1. Eighty patients (44 male and 36 female) with a mean age of 66.8±11.3 years were enrolled in the study; 20 in each group for CKD stage (2, 3a, 3b, and 4). The majority of the patients (56.3%) had only completed primary school, and most of them were either never smokers (43.8%) or ex-smokers (40%). The most common comorbidity was arterial hypertension (88.8%), followed by diabetes mellitus (57.5%). Regarding their residency, fifty-six patients (70%) reported the city (Thessaloniki) as the main place of residence, while 36 (42%) of participants use the stairs daily instead of using the elevator for access to their home.

**Table 1:** Baseline demographic, clinical and residential characteristics of the study participants Continuous variables are reported as mean±standard deviation (SD) or median [interquartile range] according to the normality of distribution tested with the Shapiro-Wilk test. Categorical variables are presented as absolute frequencies and percentages (n, %). CKD - chronic kidney disease; COPD - chronic obstructive pulmonary disease

Parameter	N (%)
N	80
Age (years)	66.8 ± 11.3
Gender (n, %)	
Male	44 (55%)
Female	36 (45%)
Education level	
Primary school	45 (56.3%)
Middle school	7 (8.8%)
High school	17 (21.3%)
Higher education - university	6 (7.5%)
Higher education - technological institute	4 (5%)
Stages of CKD	
2	20 (25%)
3A	20 (25%)
3B	20 (25%)
4	20 (25%)
Smoking history	
Never smoker	35 (43.8)
Current smoker	13 (16.3)
Ex-smoker	32 (40%)
Medical history (comorbidities)	
Diabetes mellitus	46 (57.5)
Arterial hypertension	71 (88.8)
Coronary artery disease	16 (20%)
Peripheral artery disease	12 (15%)
Previous stroke	5 (6.3%)
Arrhythmia	7 (8.8)
COPD	3 (3.8)
Autoimmune disorder	3 (3.8)
Cancer	8 (10)
Place of Residence	
City	56 (70%)
Town	9 (11.3%)
Village	15 (18.8%)
Type of home	
Apartment	59 (73.8%)
Detached house	21 (26.2%)
Access to home	
Elevator	42 (52.5%)
Stairs	36 (45%)
Garden in the house	24 (30)
Use of bike	1 (1.3%)

Physical activity levels of patients

Data on physical activity are presented in Table 2; both physical activity intensity, expressed as calculated moderate intensity metabolic equivalents (METS) minutes/wk, and physical activity class (high, moderate, and low) are presented for the total population and across CKD stages. The patient population is very inactive, with only 10% presenting with high levels of physical activity, while no significant differences were noted between disease stages (p=0.567 for high-intensity, p=0.588 for moderate-intensity, and p=0.109 for low-intensity physical activity; Table 2). Regarding previous exposure to exercise, only 11 patients (13.8%) reported that sometime in the past, they were systematically involved with a sport, but none of them at the time of the study.

**Table 2:** Physical activity intensity based on calculated METS and category of physical activity, in total patient population and across CKD stages CKD - chronic kidney disease; IPAQ: International Physical Activity Questionnaire; METS - moderate intensity metabolic equivalents

Physical activity intensity (METS min/week)	Value	p-value (for CKD groups comparison)
Total high, median (min-max)	0 (0 - 7200)	
CKD stage 2	0 (0-1920)	
CKD stage 3a	0 (0-7200)	0.567
CKD stage 3b	0 (0)	
CKD stage 4	0 (0)	
Total moderate, median (min - max)	0 (0 - 5880)	
CKD stage 2	0 (0 - 1500)	
CKD stage 3a	0 (0 - 4800)	0.588
CKD stage 3b	0 (0 - 5880)	
CKD stage 4	0 (0 - 5880)	
Total low, median (min - max)	354.8 (0 - 4851)	
CKD stage 2	478.5 (0 - 2772)	
CKD stage 3a	321.8 (0 - 1617)	0.109
CKD stage 3b	536.3 (0 - 4851)	
CKD stage 4	264 (0 - 1732.5)	
IPAQ total, median (min - max)	743 (0 - 12248)	
Level of physical activity		
High, % (n)	10 (8)	
Moderate, % (n)	41.3 (33)	
Low, % (n)	48.7 (39)	

Physical activity levels of carers

Eighty carers who lived in the same environment as each patient were included; 63.7% were female, and 36.3% were male. The carers, aged 61 ± 14.1 years old, were slightly younger than the patients (67.8 ± 11.4, p= 0.001). Forty-nine (61.3%) of carers were the patient's spouse, followed by their child (n=17, 21.3%) and a close friend (n=14, 17.5%). Among carers, 17 (21.3%) were categorized in high physical activity class, 32 (40%) in moderate, and 31 (38.8%) in low. The physical activity of the carers was significantly higher than this of the patients, regarding moderate intensity METS (p=0.03), and there was a trend towards higher vigorous-intensity METS (p=0.053); low-intensity METS and total METS were similar in the two groups (p=0.076 and p=0.083, correspondingly). Moreover, no significant correlation was noted between the IPAQ score of the patient and of the carers (p=0.702).

Exercise capacity

All patients were able to complete the 30-STS test without significant desaturation or blood pressure drop. The number of repetitions significantly decreased from CKD stage 2 to stage 4 (Table 3). A second analysis also indicated a significant decrease in STS repetitions with increasing age (Table 3). When these parameters were entered into the linear regression analysis, both the age category (standardized beta coefficient -0.353, p=0.001) and CKD stage (standardized beta coefficient -0.320, p=0.002) remained independent predictors of STS repetitions. No correlations were noted between STS repetitions and physical activity measures in CKD patients.

**Table 3:** Exercise capacity, as measured by number of repetitions in 30sec Sit-to-Stand test, in total patient population, across CKD stages and across age groups CKD - chronic kidney disease

	Mean number of repetitions ± SD	p-value
Total patient population	11.9 ± 4.1	
CKD stages		
CKD stage 2	14.2 ± 2.9	
CKD stage 3a	12.6 ± 5.4	0.008
CKD stage 3b	10.4 ± 3.3	
CKD stage 4	10.5 ± 3.5	
Age group		
Up to 60	14.1 ± 4.4	
60-64	12.5 ± 3.8	
65-69	12.9 ± 3.2	0.023
70-74	10.8 ± 5	
75-79	10.6 ± 3.1	
80 and above	9.7 ± 3.9	

Effect of residential environment and sociodemographic factors on physical activity

All data regarding differences in physical activity, according to residential factors are presented in Table 4. Patients who lived in a detached house were significantly more active compared to the ones living in an apartment (p=0.013 for moderate intensity and p=0.017 for high-intensity physical activity). Likewise, the ones who lived in a house with a garden presented higher moderate (p=0.019) and vigorous (p=0.022) physical activity, while the ones with stair access to the house also presented higher levels of moderate intensity activity (p=0.037). There was also a trend for patients living in a village to be more active. No difference was noted for any residential factor regarding low-intensity (walking) activity.

No differences in physical activity levels were noted based on gender, age group, education level, or smoking history (data not shown).

**Table 4:** Physical activity intensity and residential parameters among CKD patients CKD; chronic kidney disease; PA -physical activity

	High-intensity PA (METS min/week)	Moderate-intensity PA (METS min/week)	Low-intensity PA (METS min/week)
Place of residence			
City	0 (0 -7200)	0 (0 - 4800)	363 (0 - 2772)
Town	0 (0 - 120)	0 (0 - 5880)	330 (0 - 4851)
Village	0 (0 - 1920)	480 (0 - 5880)	462 (0 - 1732.5)
P for comparisons	0.509	0.050	0.747
Type of home			
Apartment	0 (0 - 0)	0 (0 - 5880)	404.3 (0 - 4851)
Detached house	0 (0 - 7200)	480 (0 - 5880)	247.5 (0 - 1732.5)
P for comparisons	0.017	0.013	0.157
Presence of garden			
Yes	0 (0 - 7200)	360 (0 - 5880)	297 (0 - 1732.5)
No	0 (0)	0 (0 - 5880)	404.5 (0 - 4851)
P for comparisons	0.022	0.019	0.156
Access to home			
Stairs	0 (0 - 7200)	50 (0 - 5880)	412.5 (0 - 4851)
Elevator	0 (0)	0 (0 - 5880)	354.8 (0 - 2772)
P for comparisons	0.124	0.037	0.650

## Discussion

This cross-sectional study evaluated the physical activity level in patients with CKD of different stages and investigated, for the first time, its potential association with the physical activity of carers and with patients' housing environment. No significant differences in physical activity class or intensity were noted between patients across CKD stages 2-4, although exercise capacity significantly decreased with disease severity. Carers' physical activity level was significantly higher and not correlated to one of the patients; however, physical activity among patients differed according to several residential characteristics, such as area of living and type of housing.

Patients with CKD present extremely low physical activity levels; previous observational studies have shown that patients with CKD engage in physical activity only about nine days per month, with 45% of those with end-stage kidney disease reporting no exercise at all [18]. A prospective study with 330 non-dialysis-dependent CKD patients (stages 1-4) showed that the majority of patients with CKD show only mild intensity physical activity (i.e., walking), regardless of the stage of the disease, with activity levels falling well below the recommended guidelines; this physical inactivity was suggested to be one of the most common components of frailty [25]. Our study confirms these findings; physical activity was very low, regardless of the patient's disease stage. Only 10% of participants presented with health-enhancing physical activity levels, while approximately 49% were engaged only in low-intensity activities, such as walking. Age was not associated with weekly min METS in our population. However, the fact that patients are elderly, with a mean age of 67 years, and presented with several comorbidities might partially explain their inactivity. Nevertheless, their clinical and demographic characteristics are indifferent to other similar studies regarding CKD populations [26].

The physical activity of carers differed significantly from the patients, especially regarding moderate-intensity activity, and no correlation was noted between the two groups (patients and carers) regarding weekly min METS of physical activity. Nevertheless, activity levels were also very low among carers and much lower than those recommended by the World Health Organization [27], as only 17% of them presented with health-enhancing physical activity levels. Previous studies indicated that factors related to the social environment may influence the movement behavior of patients with chronic disorders [28]. A carer is a key member of the immediate social environment of the patient. To what extent the carer's beliefs and attitudes towards physical activity may induce similar attitudes from the patient is still unknown. However, social support and social environment are currently identified as key elements for an effective intervention to reduce sedentary time in the literature [29,30]. Thus, any potential interventions targeting the increase of physical activity among CKD patients may be more effective in terms of adherence and behavioral change if it includes the patient's carer, for whom inactivity is also a major issue. Nevertheless, whether this approach, which has been previously applied in other chronic disorders [28], could also be effective in patients with CKD is a hypothesis that needs to be further investigated with larger cohort studies in the future.

Although physical activity was very low irrespective of CKD stage, the 30-STS significantly decreased with disease severity and increased age. The number of STS repetitions is associated with physical functioning and skeletal muscle (quadriceps) strength, and cachexia is a common feature in patients with CKD. Our results are in agreement with literature, as the excellent association between deterioration of renal function and decrease in STS performance has been previously proven; for every 1 mL/min/1.73 m^2^ drop in GFR, the odds are 1.5 times higher that the patient would not be able to rise unassisted from a chair once (STS1) [31,32]. The discrepancy between physical activity, which is very low from the very early stages, and exercise capacity, which decreases with disease progression, indicates that disease per se and its complications are not the main causes of an inactive lifestyle. This is why rehabilitation programs, although they are very effective in increasing muscle strength and exercise capacity among patients with several chronic diseases, may not result in increased long-term daily physical activity unless they induce behavioral changes and healthy lifestyle habits [33,34].

Residential factors were the only parameters that were associated with increased daily physical activity in our study. Living in a detached house (compared to an apartment), having a garden, and accessing the house by stairs (compared to the elevator) were all associated with higher-intensity physical activity. There was also a trend for higher physical activity among patients who lived in a village compared to a town or city. Previous studies in adults have indicated that micro- and macroenvironment in the area of living seem to be an important determinant of physical activity, with high street connectivity, high neighborhood walkability, availability and proximity of transport system being positively associated with daily physical activity [20,35,36]. Although housing characteristics are an important part of this microenvironment, their specific role in physical activity traits has been little investigated in patients with chronic diseases. To the authors' knowledge, this is the first time that such an association has been evaluated for patients with CKD.

This study carries several strengths. All data were recorded during a baseline visit, and physical activity information referred to the preceding seven days, thus minimizing recall bias. Moreover, a specific strategy has been applied in order for all CKD stages to be equally represented so that the impact of disease severity on physical activity levels could be accurately evaluated.

A limitation of the study would be that no activity monitor was used to evaluate the number of steps or other similar measures of physical activity. Nevertheless, IPAQ is a valid tool that has been repeatedly used in the literature for the assessment of physical activity in several healthy and clinical populations [22,37]. Moreover, the study sample size is somehow low compared to other similar studies in the field; however, a large age span and all CKD stages are represented in the patient population for both genders, increasing the accuracy of the results. 

## Conclusions

In conclusion, physical activity in patients with CKD was very low from the early stages, without further decreasing with disease progression, although exercise capacity deteriorated from stage 2 to 4. Carers presented with significantly higher physical activity intensity than patients, but they were also very inactive, while residential characteristics were the only factors associated with physical activity levels among patients. Since social support and proper microenvironment may induce physical activity in patients with chronic disorders, targeted, personalized interventions are needed based on each patient's barriers and facilitating factors. In this course, carers could be a valuable alley, providing they also embrace an active lifestyle. Future studies are needed in order to identify whether tailored interventions targeting the patient-carer couple and introducing exercise within their microenvironment may maximize adherence to healthier, more active daily living. 
